# Coumarins from *Magydaris pastinacea* as inhibitors of the tumour-associated carbonic anhydrases IX and XII: isolation, biological studies and in silico evaluation

**DOI:** 10.1080/14756366.2020.1713114

**Published:** 2020-01-17

**Authors:** Benedetta Fois, Simona Distinto, Rita Meleddu, Serenella Deplano, Elias Maccioni, Costantino Floris, Antonella Rosa, Mariella Nieddu, Pierluigi Caboni, Claudia Sissi, Andrea Angeli, Claudiu T. Supuran, Filippo Cottiglia

**Affiliations:** a Department of Life and Environmental Sciences, University of Cagliari, Cagliari, Italy; b Department of Chemical and Geological Sciences, University of Cagliari, Cagliari, Italy; c Department of Biomedical Sciences, University of Cagliari, Cittadella Universitaria di Monserrato, Cagliari, Italy; d Department of Pharmaceutical and Pharmacological Sciences, University of Padova, Padova, Italy; e NEUROFARBA Department, Sezione di Scienze Farmaceutiche e Nutraceutiche, Università degli Studi di Firenze, Firenze, Italy

**Keywords:** Carbonic anhydrase, coumarin, inhibitor, natural product, *Magydaris pastinacea*

## Abstract

In an *in vitro* screening for human carbonic anhydrase (hCA) inhibiting agents from higher plants, the petroleum ether and ethyl acetate extracts of *Magydaris pastinacea* seeds selectively inhibited hCA IX and hCA XII isoforms. The phytochemical investigation of the extracts led to the isolation of ten linear furocoumarins (**1**–**10**), four simple coumarins (**12**–**15**) and a new angular dihydrofurocoumarin (**11**). The structures of the isolated compounds were elucidated based on 1 D and 2 D NMR, MS, and ECD data analysis. All isolated compounds were inactive towards the ubiquitous cytosolic isoform hCA I and II (*K*_i_ > 10,000 nM) while they were significantly active against the tumour-associated isoforms hCA IX and XII. Umbelliprenin was the most potent coumarin inhibiting hCA XII isoform with a *K*_i_ of 5.7 nM. The cytotoxicity of the most interesting compounds on HeLa cancer cells was also investigated.

## 1. Introduction

Coumarins occur as secondary metabolites in many plant species, but above all, in Apiaceae, Rutaceae, Asteraceae and Fabaceae. Natural coumarins have attracted considerable attention because of their wide range of biological activities including anti-HIV^1^, antimicrobial^2^, anticancer^3^^,^^4^, anticoagulant^5^, antioxidant^6^, and antiinflammatory^7^ properties. The discovery of the natural coumarin 6-(1S-hydroxy-3-methylbutyl)-7-methoxy-2H-chromen-2-one as inhibitor of carbonic anhydrase (CA, EC 4.2.1.1) extended the spectrum of the pharmacological activity of coumarins also towards this class of enzymes^8^.

CAs are ubiquitous metallo-enzymes, which are widely present in prokaryotes and eukaryotes. Sixteen different human (h) CA isoforms (I–XV, VA and VB) are present in mammals. These enzymes catalyse a very simple but essential physiological reaction of the life cycle of many organisms, the reversible hydration of carbon dioxide to bicarbonate and protons. As a consequence, CAs participate in various physiological and pathological processes and the deregulation of CA activity is associated with disorders and diseases such as cancer^9^, glaucoma^10^, epilepsy^11^ and obesity^12^. Not surprisingly, hCA inhibitors have been intensively studied and several are in clinical use for diverse pathologies^10^^,^^13^^,^^14^. However, the systemic administration of hCA inhibitors (hCAIs) causes a wide range of side effects due to the lack of isozyme selectivity that leads to inhibition of the ubiquitous hCA I and II isoforms. Accordingly, there is a continuous search of isoform-selective CAIs which should not inhibit the off-target CAI and II. The transmembrane hCA IX and XII are well-known tumour-associated isoforms and are overexpressed in many hypoxic tumours showing a restricted expression in normal tissues. In particular, under hypoxic conditions CA IX and XII decrease extracellular pH and promote tumour cell survival and invasion in hypoxic microenvironments^15^^,^^16^. For this reason, hCA IX and XII are attractive anticancer drug targets. Few natural coumarins have been evaluated as inhibitors of hCA IX and XII isoforms^8^^,^^17^^,^^18^ but some of them were found active in the nanomolar range^18^.

Continuing our search for biologically active secondary metabolites from Sardinian higher plants^19^^,^^20^, we have found that the petroleum ether and ethyl acetate extracts of *Magydaris pastinacea* seeds exhibited potent inhibitory activity against hCA IX and XII. As a consequence, in order to identify the active compounds, a phytochemical investigation of the extracts was performed. *Magydaris pastinacea* (Lam.) Paol. (Apiaceae) [syn.: *Magydaris tomentosa*] is a plant that spontaneously grows in few regions of Mediterranean area such as Sicily, Sardinia, Corse, Baleares and North Africa. Previous studies on the rhizomes of *M. pastinacea* revealed as main metabolites coumarin and furocoumarin glucosides with inhibitory activity on platelet aggregation^21^. Furthermore, coumarins isolated from the flowers of the same plant showed antibacterial and anticoagulant activities^22^ while a more recent work on the petroleum ether extract of flowers reported the cytotoxicity of furocoumarins and simple coumarins^23^.

## 2. Materials and methods

### 2.1. General experimental procedures

Optical rotations were measured in CHCl_3_ or MeOH at 25 °C using a Perkin-Elmer 241 polarimeter. Circular dichroism spectra were recorded on a JASCO J-810 spectropolarimeter equipped with a Peltier temperature controller using a 10 mm path-length cell. All measurements were performed in methanol at compound concentration of 300 µM. Each reported spectrum represents the average of 3 scans recorded with 1-nm step resolution. Observed ECD signals were converted to molar ellipticity [Θ] = deg × cm^2^ × dmol^−1^. UV spectra were recorded on a GBC Cintra 5 spectrophotometer. NMR spectra of all isolated compounds were recorded at 25 °C on Unity Inova 500NB high-resolution spectrometer (Agilent Technologies, CA, USA) operating at 500 MHz for ^1^H and 100 MHz for ^13^C, respectively. Spectra were measured in CDCl_3_ and CD_3_OD and referenced against residual non-deuterated solvents. HRESIMS were measured on an Agilent 6520 Time of Flight (TOF) MS instrument. Column chromatography was carried out under TLC monitoring using silica gel (40–63 μm, Merck), and Sephadex LH-20 (25–100 μm, Pharmacia). For vacuum-liquid chromatography (VLC), silica gel (40–63 μm) (Merck) was used. TLC was performed on silica gel 60 F_254_ or RP-18 F_254_ (Merck). LiChrolut RP-18 (40–63 μm) 500 mg, 3 mL (Merck) solid phase extraction (SPE) cartridges were also used. Semi-preparative HPLC was conducted by means of a Varian 920 LH instrument fitted with an autosampler module with a 1000 µL loop. The peak purities were monitored using a dual-wavelength UV detector settled at 254 and 360 nm. The columns were a 250 × 10 mm Spherisorb silica, particle size 5 μm (Waters) and a 300 7.5 mm Polymeric Reversed Phase (PLRP-S 100 Å), particle size 8 μm (Varian).

### 2.2. Plant material

The seeds of *M. pastinacea* were collected in July 2017 at Siniscola (Nuoro), Sardinia, Italy. The plant material was identified by Prof. Marco Leonti (University of Cagliari, Department of Biomedical Sciences). A voucher specimen (No. 0485) was deposited in the Herbarium of the Department of Life and Environmental Science, Drug Sciences Section, University of Cagliari.

### 2.3. Extraction and isolation

Air-dried and powdered seeds of *M. pastinacea* (720 g) were ground and extracted with petroleum ether (3.5 L) by percolation at room temperature to give 77.6 g dried extract. The remaining plant material was then extracted with EtOAc (3 L), giving 42 g dried extract.

An aliquot (20 g) of the petroleum ether extract was subjected to Vacuum Liquid Chromatography (VLC) (silica gel, 90 g, 40–63 μm) using a step gradient of *n*-hexane/ethyl acetate (9:1–0:10, 500 mL each) to yield 24 fractions. Based on the TLC similarities, identical fractions were combined to give a total of eight fractions (F1–F8). Fraction F3 (0.98 g) was separated by column chromatography (CC) over silica gel using toluene/CH_2_Cl_2_ (7:3) as eluent to isolate compound **15** (96 mg). Fraction F4 (1.55 g) was chromatographed by CC over silica gel using hexane-EtOAc (1:9) as eluent giving compound **12** (330 mg). Fraction F7 (1.04 g) was purified by CC over silica gel, using CH_2_Cl_2_-EtOAc (9.5:0.5) as eluent to give eight subfractions (F7.1–F7.8). F7.2 (11 mg) was subjected to chromatography by Sephadex LH (MeOH) yielding compound **1** (3 mg). Subfraction F7.3 (110 mg) was further subjected to CC over silica gel using CH_2_Cl_2_ as eluent to give compound **2** (2.5 mg) and a white solid (90 mg). The obtained solid was purified further by PLRP HPLC using acetonitrile : H_2_O (6:4, flow 2.5 mL/min) as eluent to give compound **2** (1.5 mg, *t*_R_ 11.2 min) and compound **3** (1.2 mg, *t*_R_ 13 min). Subfractions F.7.6 and F7.8 were purified by Sephadex LH-20 (MeOH) yielding compounds **8** (38.9 mg) and **13** (170 mg), respectively. Fraction F8 (800 mg), after purification by CC over Sephadex LH-20 (MeOH), furnished compound **13** (670 mg).

An aliquot (16.6 g) of the EtOAc extract was subjected to Vacuum Liquid Chromatography (VLC) (silica gel, 80 g, 40–63 μm) using a step gradient of *n*-hexane/ethyl acetate/MeOH (9:0 : 0–0:1: 9, 500 mL each) to yield 38 fractions. Based on the TLC similarities, identical fractions were combined to give a total of ten fractions (F1–F10). An aliquot (0.5 g) of F3 (3.74 g) was chromatographed over silica gel using CH_2_Cl_2_ as eluent, giving compound **6** (61.2 mg) and compound **7** (4.7 mg). An aliquot (50 mg) of F4 (1.56 g) was purified and an impure compound (12 mg) that was purified by PLRP HPLC using acetonitrile : H_2_O (7:3, flow 2 mL/min) as eluent to give compound **2** (3.9 mg, *t*_R_ 8.5 min) and compound **1** (2.1 mg, *t*_R_ 14.2 min). Fraction F6 (0.18 g) was subjected to CC over silica gel using CH_2_Cl_2_ : EtOAc (7.5:2.5) as eluent, giving eight subfractions (F6.1–F6.8). Fraction F6.3 (41 mg) was further chromatographed by PLRP HPLC, using acetonitrile : H_2_O (5:5, flow of 2 mL/min) as eluent, to give compounds **4** (1 mg, *t*_R_ 13.2 min) and **5** (1.3 mg, *t*_R_ 15.2 min). Fraction F6.5 (6.5 mg) was separated by PLRP HPLC, using acetonitrile : H_2_O (5:5, flow of 2 mL/min) as eluent, to give compounds **10** (0.9 mg, *t*_R_ 8.5 min) and **11** (2.8 mg, *t*_R_ 7.5 min). An aliquot (40 mg) of F7 (70 mg) was chromatographed on CC over Sephadex LH-20 (MeOH) to furnish an impure compound (16 mg) that was further purified by PLRP HPLC using acetonitrile : H_2_O (5:5, flow 2 mL/min) as eluent, to give compound **11** (1.3 mg, *t*_R_ 11.0 min), compound **10** (1 mg, *t*_R_ 13 min), compound **4** (1.2 mg, *t*_R_ 14.5 min) and compound **5** (3.6 mg, *t*_R_ 15.5 min). Fraction F8 (165 mg) was subjected to CC over Sephadex LH (MeOH), yielding four subfraction (F8.1–F8.4). Subfraction F8.2 (34.8 mg) was purified by CC over Sephadex LH-20, followed by PLRP HPLC using acetonitrile : H_2_O (4:6, flow 2.0 mL/min) as eluent, to give compound **14** (3.8 mg, *t*_R_ 13.5 min). Subfraction F8.3 (42.2 mg) was purified by PLRP HPLC using acetonitrile : H_2_O (4:6, flow 2.0 mL/min) as eluent, to give compound **9** (5.1 mg, *t*_R_ 9.5 min). Subfraction F8.4 (30 mg), was further subjected to CC over Sephadex LH-20 (MeOH) to yield compound **9** (2.7 mg).

*Magydarin* (**11**): white powder; [α]^25^_D_ + 96.3 (*c* 0.05, CH_2_Cl_2_); UV (MeOH) *λ*_max_ (log ε) 348 (8.5) nm; ECD (300 µM, MeOH) *λ* (Δε) 348 (+2950) nm; ^1^H (CDCl_3_, 500 MHz) and ^13 ^C (CDCl_3_, 100 MHz) NMR, see Table 1; HRTOFESIMS *m/z* 277.1078 [M + H]^+^ (calcd for C_15_H_16_O_5_, 277.1076).

**Table 1.** ^1^H NMR and ^13^C NMR Spectroscopic Data for Compound **11** (CDCl_3_, *δ* in ppm).

Compound **11**		
Position	δ_C_, type	δ_H_ (*J* in Hz)
2	161.2, C	
3	112.8, CH	6.23, d (9.5)
4	143.7, CH	7.59, d (9.5)
5	109.4, CH	6.77, s
6	141.8, C	
7	146.1, C	
8	115.5, C	
9	152.5, C	
10	112.8, C	
2′	92.2, CH	4.86, *t*, (9)
3′	28.2 CH_2_	3.36, dd (1.5, 9)
4′	71.8, C	
5′	24.1 CH_3_	1.25, s
6′	26.1, CH_3_	1.40, s
OCH_3_	56.4, CH_3_	3.91, s

### 2.4. Semi-Synthesis of (+)-heraclenol acetate (10)

To a solution of (−)-heraclenol (**9**) (38.9 mg, 0.13 mmol) in pyridine (2 mL) acetic anhydride (0.012 mL, 0.13 mmol) was added dropwise and left at room temperature for 48 h. The crude product was dried and purified by CC (Sephadex LH-20), using MeOH as eluent to provide 24.9 mg of (+)-heraclenol acetate (**10**)^24^.

### 2.5. Molecular modelling

The ligands were built within the Maestro platform. The most stable conformation has been determined by molecular mechanics conformational analysis performed with Macromodel software version 9.2^25^ using the Merck Molecular Force Fields (MMFFs)^26^ and GB/SA water implicit solvation model^27^, Polak-Ribier Conjugate Gradient (PRCG) method, 5000 iterations and a convergence criterion of 0.05 kcal/(mol Å). All the other parameters were left as default.

The coordinates for CA enzyme were taken from the RCSB Protein Data Bank^28^ (PDB code 4ww8)^29^. The protein was prepared by using the Maestro Protein Preparation Wizard. Original water molecules were removed. Molecular docking studies were performed using the QMPL workflow protocol. Grids were defined around the refined structure by centreing them on the co-crystallized ligand. The other settings were left as default.

In order to better take into account the induced fit phenomena, the most energetically favoured generated complexes were fully optimised with the OPLS2005 force field in GB/SA implicit water^30^. The optimisation process was performed setting 10,000 steps interactions up to the derivative convergence criterion equal to 0.05 kJ/(mol*Å). The resulting complexes were considered for the binding modes graphical analysis with Pymol and Maestro.

### 2.6. Biological activity

#### 2.6.1. Carbonic anhydrase inhibition assay

An Applied Photophysics stopped-flow instrument has been used for assaying the CA catalysed CO_2_ hydration activity^31^. Phenol red (at a concentration of 0.2 mM) was used as indicator, working at the absorbance maximum of 557 nm, with 20 mM Hepes (pH 7.5) as buffer and 20 mM Na_2_SO_4_ (for maintaining constant the ionic strength), following the initial rates of the CA-catalysed CO_2_ hydration reaction for a period of 10–100 s. The CO_2_ concentrations ranged from 1.7 to 17 mM for the determination of the kinetic parameters and inhibition constants. For each inhibitor, at least six traces of the initial 5–10% of the reaction have been used for determining the initial velocity. The uncatalyzed rates were determined in the same manner and subtracted from the total observed rates. Stock solutions of inhibitor (0.1 mM) were prepared in distilled–deionized water, and dilutions up to 0.01 nM were done thereafter with the assay buffer. Inhibitor and enzyme solutions were preincubated together for 6 h at room temperature prior to assay in order to allow for the formation of the E–I complex. The inhibition constants were obtained by nonlinear least-squares methods using PRISM 3 and the Cheng–Prusoff equation, as reported earlier^32^^,^^33^, and represent the mean from at least three different determinations. All CA isoforms were recombinant ones obtained in-house as reported earlier^34^.

#### 2.6.2. Cytotoxic assay

##### 2.6.2.1. Cell culture

Human carcinoma HeLa cell line was obtained from the American Type Culture Collection (ATCC, Rockville, MD). Cells were grown in Dulbecco’s modified Eagle’s medium (DMEM) with high glucose, supplemented with 10% foetal calf serum (FCS), penicillin (100 units/mL)–streptomycin (100 μg/mL), and 2 mM L-glutamine in a 5% CO_2_ incubator at 37 °C. Subcultures of the HeLa cells were grown in T-75 culture flasks and passaged with a trypsin-EDTA solution. Cell culture materials were purchased from Invitrogen (Milan, Italy).

##### 2.6.2.2. MTT assay

The *in vitro* cytotoxic effect of coumarins **5**, **9**–**12**, **15** was evaluated in cancer HeLa cells by the MTT (3-(4,5-dimethylthiazol-2-yl)-2,5-diphenyltetrazolium bromide) reduction assay^35^. Cancer cells were seeded in 96-well plates (density of 3 × 10^4^ cells/mL) in 100 μL of medium and cultured for 48 h (80% of cell confluence). Cells were subsequently incubated for 48 h with various concentrations (0.1–100 μM, dissolved in DMSO) of coumarins in culture medium (treated cells). Treated cells were compared for viability to untreated cells (control cells) and vehicle-treated cells (incubated for 48 h with an equivalent volume of DMSO; the maximal final concentration was 1%). After the cell medium removing and washing, cells were subjected to the MTT test^35^. After incubation (3 h), colour development was measured at 570 nm with an Infinite 200 auto microplate reader (Infinite 200, Tecan, Austria); the absorbance is proportional to the number of viable cells. Two independent experiments were performed. The results were calculated as the percentage of cell viability in comparison with non-treated control cells and expressed as IC_50_ value (the concentration of compound that reduces the cell viability to 50%).

## 3. Results and discussion

### 3.1. Isolation and characterisation

**Figure 1.**
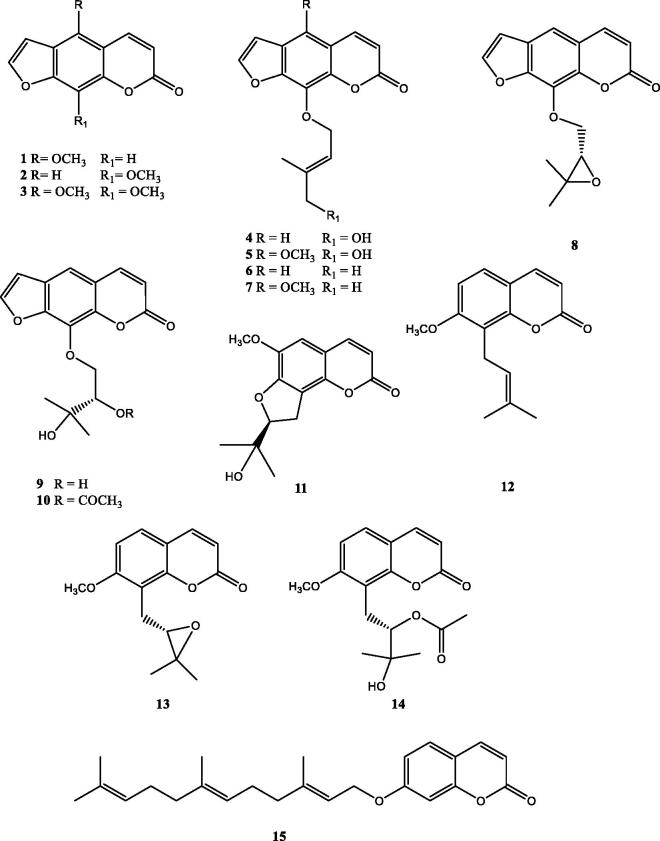
Structures of the isolated coumarins.

The petroleum ether and ethyl acetate extracts of *M. pastinacea* showed high potency to inhibit hCA IX and XII isoforms (Table 2) and were therefore subjected to fractionation by silica gel vacuum-liquid chromatography (VLC), column chromatography (silica gel and Sephadex LH 20) and semi-preparative HPLC (Polymeric RP-HPLC) to give one new angular dihydrofurocoumarin (**11**) along with ten linear furocoumarins (**1–10**) and four simple coumarins (**12**–**15**) (Figure 1).

**Table 2.** Inhibition data towards hCA I, II, IX, and XII of compounds **1**–**15.**

	*K*_i_ (nM)*			
Compound/extract	hCA I	hCA II	hCA IX	hCA XII
Petroleum ether	>100^a^	>100^a^	20^a^	0.8^a^
Ethyl acetate	>100^a^	>100^a^	1.74^a^	0.5^a^
**1**	>10,000	>10,000	1953	855.1
**2**	>10,000	>10,000	194.8	876.3
**3**	>10,000	>10,000	159.8	590.1
**4**	>10,000	>10,000	2339	550.0
**5**	>10,000	>10,000	1501	63.5
**6**	>10,000	>10,000	221.4	832.9
**7**	>10,000	>10,000	201.9	786.9
**8**	>10,000	>10,000	162.5	835.6
**9**	>10,000	>10,000	27.5	813.8
**10**	>10,000	>10,000	192.5	>10,000
**11**	>10,000	>10,000	150.9	623.0
**12**	>10,000	>10,000	2471	74.5
**13**	>10,000	>10,000	1888	>10,000
**14**	>10,000	>10,000	>10,000	290.9
**15**	>10,000	>10,000	266.4	5.8
**AAZ**	250.0	12.1	25.8	5.7

^a^ Data expressed in ng/mL.

^*^ Mean from three different assays, by a stopped flow technique (errors were in the range of ±5–10% of the reported values).

**Figure 2.**
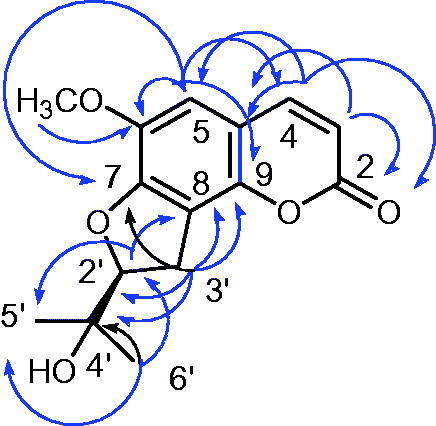
Main HMBC correlations of compound **11**.

The HR-ESIMS of compound **11** showed a molecular ion at *m/z* 277.1078 (M + H)^+^ which is in accordance with the molecular formula C_15_H_16_O_5_ (calcd. 277.1076). The ^1^H NMR spectrum of compound **11** showed two doublets at 6.23 (H-3, *J =* 9.5 Hz) and 7.59 (H-4, *J =* 9.5 Hz) ppm and a singlet at 6.77 ppm characteristic of a coumarin nucleus substituted at C-6, C-7 and C-8 (Table 1). In the high field spectrum region a further singlet at δ 3.91 (3H, s) was characteristic of a methoxy group, while two singlets at 1.25 (3H, s) and 1.40 (3H, s) were assigned to two tertiary methyl groups. Finally, the signals at 3.36 (2H, dd, *J* = 1.5, 9 Hz) and 4.86 (1H, t, *J* = 9 Hz) could be ascribed to a methylene and a methine group, respectively. The ^13 ^C NMR spectrum highlighted 14 carbons of which those at 161.2, 143.7 and 112.8 ppm were characteristic respectively of C-2, C-3 and C-4 of a coumarin nucleus (Table 1). The connectivity of each proton with its respective carbon has been identified through HSQC experiment. In the HMBC spectrum, the correlations between the aromatic proton at δ 6.77 (1H, s) and the carbons at 152.5, 146.1, 143. 7 and 141.8 ppm located this proton at position 5 of the coumarin system (Figure 2). This was confirmed by the fact that no cross-peak of this proton with the carbon at 143.7 ppm could be observed if it would be located at C-6, C-7 or C-8. In the same spectrum the cross-peaks between the signal at 1.25 (s) and the carbons at 26.1, 71.8 and 92.2 ppm and between the signal at 1.40 (s) ppm and the carbons at 24.1, 71.8 and 92.2 ppm, confirmed the presence of two geminal methyls.

Further correlations of the methylene protons at δ 3.36 (2H, dd, *J* = 1.5, 9 Hz) with the carbons at 71.8, 92.2, 115.5, 146.1 and 152.5 ppm fixed the 1,1-dimethyl-2-oxypropanol chain at C-8 of the coumarin nucleus. In the HMBC experiment it could be observed that the methoxyl group at 3.91 ppm was linked to the coumarin nucleus on carbon at 141.8 ppm. This data, together with the correlation between the aromatic proton at C-5 and carbon at 141.8 ppm, allowed us to place the methoxy group at C-6. The absence of further aromatic protons and the presence of only one alcohol group (δ 1.58) in the proton spectrum, suggested the cyclisation of the alcohol chain at position 7 of the coumarin ring.

HSQC, HMBC, and ROESY experiments allowed the complete assignment of all signals and the identification of the structure as reported in Figure 2.

**Figure 3.**
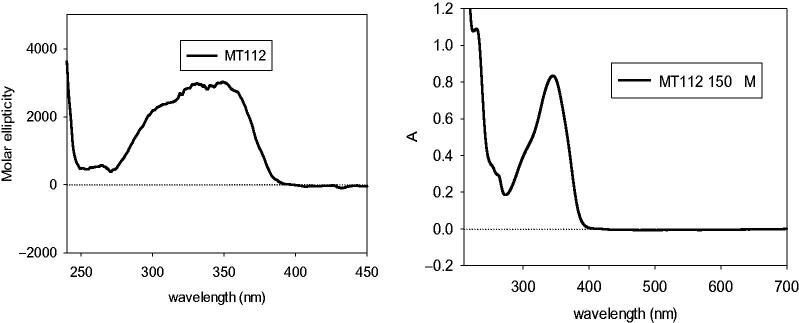
ECD (left) and UV (right) spectra of compound **11**.

The absolute configuration of the (+)-diidrofurocoumarin **11** has been established studying its Electronic Circular Dicroism (ECD) spectrum (Figure 3). The positive Cotton effect at 348 nm in the ECD spectrum was ascribed to the n → π* electronic transition of the unsaturated lactone ring, which permitted the assignment of the (2'S) absolute configuration based on the modified octant rule^36^^,^^37^. In fact, the 1-hydroxy-1-methylethyl group was located in the upper left (+) octant. Compound **11** is a previously undescribed diidrofurocoumarin and was named magydarin.

The absolute configuration of the (+)-heraclenol acetate^24^ **10** is not reported in the literature and therefore it has been established through the acetylation of the isolated (−)-heraclenol **9**, for which the S configuration has been previously assigned^38^^,^^39^. The specific optical rotation of compound **9** ([α]_D_^25^= −7.1) revealed that its absolute configuration is S^40^. Acetylation with acetic anhydride in pyridine of (−)-S-heraclenol yielded S-heraclenol acetate possessing a specific optical rotation [α]_D_^25^ +10. As consequence, the natural (+)-heraclenol acetate (**10**) must have an absolute configuration S.

Compound **4** has been identified as trichoclin by comparison with analytical and spectroscopic data reported in the literature^41^. However, trichoclin contain a double bond whose geometric configuration could not be determined by comparison with the data reported in the literature. In order to assign the *Z* or *E* configuration to the double bond, a ROESY spectrum of compound **4** was recorded. The ROESY spectrum revealed a cross-peak between the olefinic proton at δ 5.74 (1H, t, *J* = 7 Hz) and the oxymethylene protons at 5.05 (2H, d, *J* = 7 Hz) but the correlation between the proton at δ 5.74 with the methyl group at 1.87 (3H, s) ppm could not be observed. This is well suited to an *E* geometry. In fact, a 3 D molecular model of *E*-trichoclin highlighted that the distance between the olefinic proton and the oxymethylene protons was 3.2 Å, whereas the distance between the same proton and the methyl group was 4 Å. In the case of *Z* isomer, the distance of 3.9 Å between the olefinic proton and the oxymethylene protons preclude any correlation whereas the short distance (2.3 Å) of the methyl at δ 1.87 with methine at 5.74 ppm should be enough to give a cross-peak in the ROESY spectrum. Thus, compound **4** has been identified as *E*-trichoclin.

Compound **5** has been identified as 5-methoxy-trichoclin by comparison with analytical and spectroscopic data reported in the literature^41^. With respect to *trichoclin*, compound **5** contained the same chain and an additional methoxyl at position 5. As for *E*-trichoclin, the geometry of 5-methoxy-trichoclin double bond was determined by ROESY experiments. In the ROESY spectrum the cross peak between the olefinic proton at δ 5.73 (1H, t, *J* = 7 Hz) and the methylene protons at δ 4.89 (2H, d, *J* = 7.5 Hz), but not the methyl group at δ 1.87 (3H, s) could be observed. Therefore, compound **5** has been identified as *E*-5-methoxy-trichoclin.

The structures of begapten (**1**), xanthotoxin (**2**), isopimpinellin (**3**), imperatorin (**6**), phellopterin (**7**), heraclenin (**8**), (-)-heraclenol (**9**), osthol (**12**), meranzin (**13**), 2′-acetoxy-3′-hydroxy-osthol (**14**) and umbelliprenin (**15**) were deduced from the 1 D and 2 D NMR spectra and confirmed by comparison of ^1^H- and ^13 ^C NMR data with those reported in the literature^22^^,^^41–47^.

### 3.2. CA inhibition

The petroleum ether and ethyl acetate extracts together with all the isolated coumarins were tested against four hCA isoenzymes (I, II, IX and XII) (Table 2). The results showed that both extracts were selective towards the tumour-associated CA IX and CA XII since none was active against CA I and CA II up to the concentration of 100 ng/mL, whereas they were highly potent especially against CA XII. The ethyl acetate extract was more potent compared to the ether extract either against CA IX (*K*_i_ 1.74 ng/mL) either against CA XII (*K*_i_ 0.5 ng/mL). The furocoumarins **1**–**9**, **11** and coumarins **12, 15** inhibited both hCA IX and hCA XII in a nanomolar range and they were completely inactive (*K*_i_ values > 10,000 nM) against hCA I and II, thus showing high selectivity over these isoforms that are considered responsible for the side-effects of CAIs. Interestingly, meranzin (**13**) and especially the furocoumarin **10**, inhibited exclusively the IX isoform of CA with *K*_i_ ratios XII/IX > 5 and >52, respectively. On the contrary, compound **14** was a selective inhibitor of the XII isoform with a *K*_i_ ratio IX/XII > 34.

The most potent compound was umbelliprenin (**15**), with a *K*_i_ value against CA XII of 5.8 nM, comparable to that of acetazolamide (AAZ) and demonstrated high selectivity over the off-target CA I/II isoforms (*K*_i_ ratios I/XII > 1724). Also *S*-heraclenol (**9**) (*K*_i_ 27.5 nM), osthol (**12**) (*K*_I_ 75 nM) and *E*-5-methoxy-trichoclin (**5**) (*K*_I_ 63.5 nM) revealed a strong inhibitory effect towards CA IX (heraclenol) and CA XII (osthol and *E*-5-methoxy-trichoclin).

The SAR study pointed out that, in the 8-*O*-monosubstituted furocoumarins (**2**, **4**, **6**, **8**–**10**), the 2,3-dihydroxy-3-methylbutyl moiety present in *S*-heraclenol (**9**), elicited the better inhibitory profile towards hCA IX (*K*_i_ 27.5 nM). (+)-*S*-heraclenol acetate (**10**) compared to *S*-heraclenol, showed a reduction of inhibitory effects towards hCA IX and hCA XII and most interestingly, a high selectivity towards the poorly expressed in healthy tissues hCA IX over hCAXII with a selectivity index >52. Among furocoumarins **2**, **4**, **6**, **8–10**, we found that, in compounds **2**, **4**, **6**, the introduction of a methoxy group at C-5, giving compounds **3**, **5**, **7**, furnished enhanced inhibitory activity against hCA IX and hCA XII. In particular, when the methoxy derivative **5** was compared with *E*-tricholin (**4**), a 23-fold gain in potency towards the isoform CAXII was observed.

As regards 7-methoxy-8-alkyl coumarins (**12–14**), osthol (**12**) was the most potent of the hCAXII inhibitors (*K*_i_ 75 nM), highlighting the importance of a prenyl chain at C-8.

The new angular dihydrofurocoumarin, magydarin (**11**), had submicromolar inhibition of the validated cancer-associated isozymes hCA IX and XII with *K*_i_s of 150.9 and 623 nM, respectively.

### 3.3. *In silico* modelling of the interaction of compounds 9, 11, 15 with CA XII

Compounds **9, 11** and **15** are characterised by an interesting inhibitory activity towards the CA XII isoform, therefore, their mechanism of action was investigated in more details by means of computational methods. The protocol consisted of docking experiments followed by the energy minimisation of the obtained complexes. Recently, an interesting CA inhibition mechanism was reported for coumarin derivatives^8^^,^^48^. This was also found as a plausible mechanism of action of previously synthesised compounds^49^.

**Figure 4.**
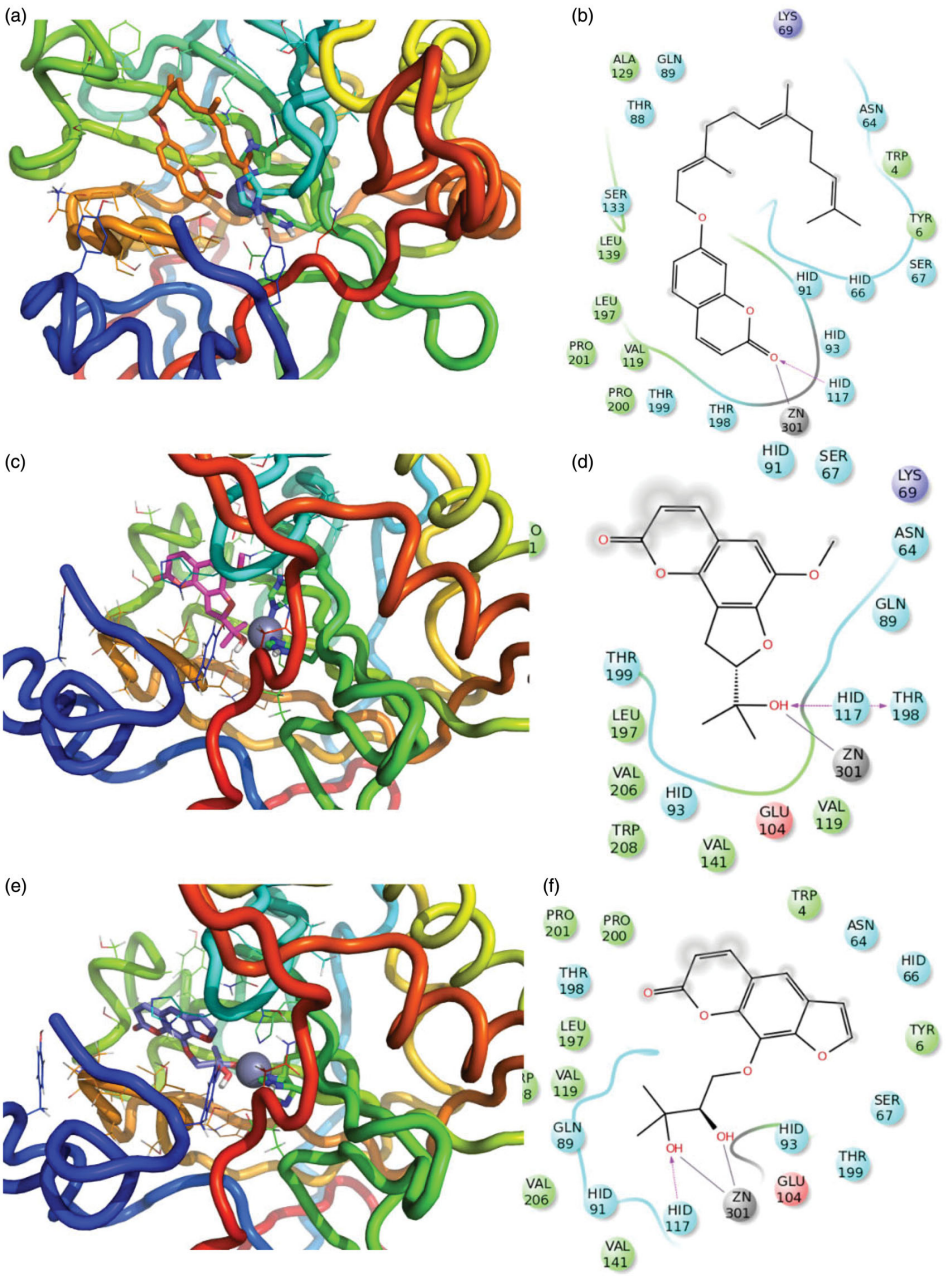
3D representation of the putative binding mode obtained by docking experiments. (a,b) CA -XII-**15** (c,d) CA -XII-**11**, (e,f) CA -XII-**9** and the relative 2D representation of the complexes stabilising interactions with the binding site residues represented with different colour depending on their chemical-physical properties: green, hydrophobic; cyan, polar; violet, positive; red, negative charged residues; grey, metal atoms. Instead, magenta arrows indicate the formation of hydrogen bond between protein and ligand, while grey lines indicate the interaction with the complexed ion.

**Figure 5.**
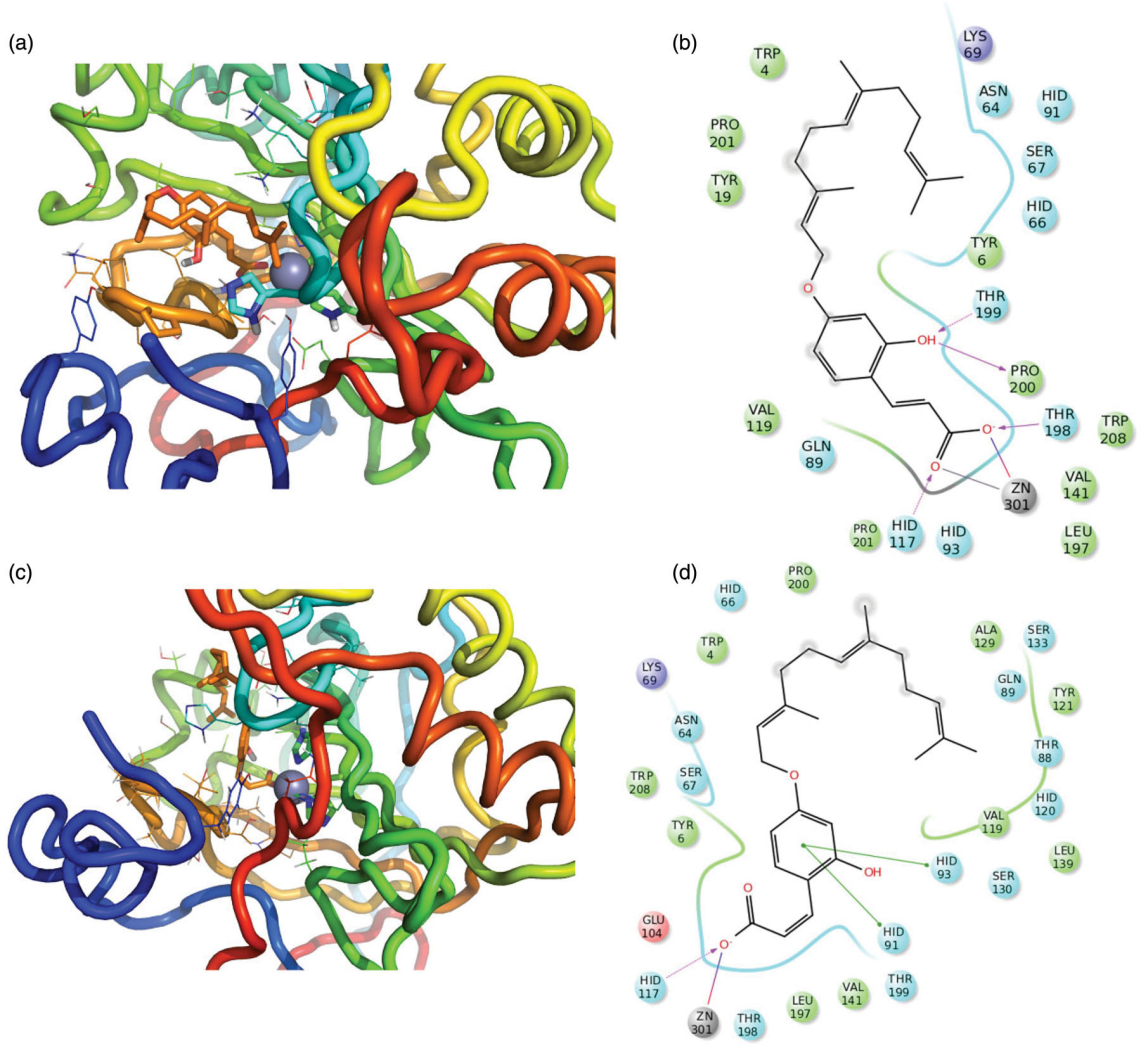
3D representation of the putative binding mode obtained by docking experiments. (a,b) CA -XII-**15-openE** (c,d) CA -XII-**15-openZ** and the relative 2D representation of the complexes stabilising interactions with the binding site residues, with the colour scheme indicated above.

Hence, the coumarin derivatives **15**, **11** and **9** were docked to understand if coumarin moiety could be hydrolysed by the Zn^2+^ activated water molecule of the enzyme cavity, which acts as a very potent nucleophile. These experiments showed that only compound **15** was able to dock the coumarin portion close enough to the Zn^2+^ (Figure 4(a)). Therefore, only this compound could be hydrolysed. Instead, the compounds **11** and **9** binding did not show the right orientation, probably due to the steric hindrance of the furocoumarin moiety (Figure 4(b,c)). Thus, both the open conformations (E/Z) of compound **15** were subjected to docking experiments in order to predict the binding mode of hydrolysed forms. It is possible to see in Figure 5 as both diastereoisomers are stabilised by several hydrogen bonds and π-π interactions in the catalytic site. The hydrophobic portion fold up and is stabilised by internal contacts and interactions with hydrophobic residues in the cavity. The predicted affinity of open compounds was estimated to be better than the closed ones.

### 3.4. Cytotoxicity of compounds 5, 9–12 and 15 towards cancer cells

On basis of the CA inhibition results, the growth inhibitory effect of the most active/interesting coumarins (**5**, **9**–**12**, **15**) on HeLa cancer cell line has been carried out (Table 3). Umbelliprenin (**15**) showed moderate cytotoxicity against HeLa cells (IC_50_ 75 μM) and this result is in accord with other studies reporting the low cytotoxicity of the compound towards the breast cancer cell lines MCF and 4T1^50^^,^^51^. However, umbelliprenin was effective to inhibit tumour growth, angiogenesis and metastasis in mammary tumour-bearing mice when intraperitoneally administrated^52^. These data were confirmed by Zhang *et al.*^53^, which provided evidence that umbelliprenin may inhibit the growth, invasion and migration of gastric cancer cells by targeting the Wnt signalling pathway, with little to no harm in the lung, heart and kidney.

**Table 3.** Cytotoxic effect of compounds **5**, **9**–**12** and **15** evaluated towards cancer HeLa cells.

Compound	**IC_50_**^a^ (µM)
**5**	>100
**9**	>100
**11**	>100
**10**	>100
**12**	98
**15**	75

^a^ Concentration of compound that reduces the cell viability to 50% measured at 48 h.

The low cytotoxicity of umbelliprenin may be related to its very high liposolubility. This hypothesis was confirmed by the fact that when umbelliprenin was encapsulated in nanoliposomes its antiproliferative effect against 4T1 cells increased by five folds^50^. Also osthol (**12**) showed only a moderate cytotoxicity with an IC_50_ of 98 μM but, as reported in previous works^54^^,^^55^, its cytotoxicity greatly depends on the cancer cell line.

Most importantly, CA IX and CA XII are overexpressed in cancer cells under hypoxic condition. Thus, the moderate antiproliferative action showed by the isolated compounds is not surprising since the assay has been carried out under normoxic conditions.

## 4. Conclusions

Fifteen coumarins were isolated from the seeds of *M. pastinacea*. Magydarin (**11**) is new to the literature, while meranzine (**13**), xanthotoxin (**2**), *E*-trichoclin (**4**), *E*-5-methoxy-trichoclin (**5**), fellopterin (**7**), (+)-*S*-heraclenol acetate (**10**) and meranzine acetate hydrate (**14**) were isolated for the first time from this plant. None of the 15 isolated coumarins have ever been evaluated for its inhibitory activity on carbonic anhydrases. All isolated compounds were inactive towards the ubiquitous cytosolic isoform hCA I and II (*K*_i_ > 10,000 nM) that are considered responsible for the side-effects of CAIs. On the contrary, they were significantly active against the tumour-associated isoforms hCA IX and XII. CA XII was inhibited with single-digit *K*_i_ by umbelliprenin (**15**) (5.7 nM) and with *K*_i_s spanning in the nanomolar range (63.5–74.5 nM) by compounds **5** and **12**. As regards the other tumour-associated isoform hCA IX, it was potently inhibited by *S*-heraclenol (**9**) (*K*_i_ = 27.5 nM) and, to a lesser extent, by the new furocoumarin magydarin (**11**). Particularly noteworthy is the selectivity of heraclenol acetate (**10**) towards hCA IX over hCAI, hCAII and hCAXII (SI > 52). As far as we know, umbelliprenin is the most potent natural coumarin CA inhibitor.

Molecular docking experiments suggested that the most potent coumarin **15** could be hydrolysed in the enzyme catalytic pocket. Furthermore, docking experiments estimated that the predicted affinity of open compounds was better than that of the closed ones. Overall the computational methods helped to rationalise the strong activity of compound **15** towards the CA-XII isoform and to suggest a plausible mechanism of action that would be further investigated to confirm it.

Future work on these compounds will attempt to synthesise analogues with lower lipophilicity to achieve a better drug-like profile.

## References

[1] Kashman Y, Gustafson KR, Fuller RW, et al. The calanolides, a novel HIV-inhibitory class of coumarin derivatives from the tropical rainforest tree, *Calophyllum lanigerum*. J Med Chem 1992;35:2735–43. 1379639 10.1021/jm00093a004

[2] Sugino A, Higgins NP, Brown PO, et al. Energy coupling in DNA gyrase and the mechanism of action of novobiocin. Proc Natl Acad Sci USA 1978;75:4838–42. 368801 10.1073/pnas.75.10.4838 PMC336216

[3] Kostova I. Synthetic and natural coumarins as cytotoxic agents. Curr Med Chem Anticancer Agents 2005;5:25–46. 10.2174/1568011053352550 15720259

[4] Pinto D, Silva A. Anticancer natural coumarins as lead compounds for the discovery of new drugs. Curr Top Med Chem 2017;17:3190–8. 29243581 10.2174/1568026618666171215095750

[5] Timson DJ. Dicoumarol: a drug which hits at least two very different targets in vitamin K metabolism. Curr Drug Targets 2017;18:500–10. 26201483 10.2174/1389450116666150722141906

[6] Kostova I. Synthetic and natural coumarins as antioxidants. Mini-Rev Med Chem 2006;6:365–74. 16613573 10.2174/138955706776361457

[7] Kirsch G, Abdelwahab AB, Chaimbault P. Natural and synthetic coumarins with effects on inflammation. Molecules 2016;21:e1322. 27706093 10.3390/molecules21101322 PMC6273422

[8] (a) Maresca A, Temperini C, Vu H, et al. Non-zinc mediated inhibition of carbonic anhydrases: coumarins are a new class of suicide inhibitors. J Am Chem Soc 2009;131:3057–62. 19206230 10.1021/ja809683v

[9] Supuran CT, Alterio V, Di Fiore A, et al. Inhibition of carbonic anhydrase IX targets primary tumors, metastases, and cancer stem cells: three for the price of one. Med Res Rev 2018;38:1799–836. 29635752 10.1002/med.21497

[10] Supuran CT, Altamimi ASA, Carta F. Carbonic anhydrase inhibition and the management of glaucoma: a literature and patent review 2013–2019. Exp Opin Ther Patents 2019;29:781–92. 10.1080/13543776.2019.1679117 31596641

[11] Thiry A, Dogné JM, Supuran CT, et al. Carbonic anhydrase inhibitors as anticonvulsant agents. Curr Top Med Chem 2007;7:855–64. 17504130 10.2174/156802607780636726

[12] De Simone G, Supuran CT. Antiobesity carbonic anhydrase inhibitors. Curr Topics Med Chem 2007;7:879–84. 10.2174/156802607780636762 17504132

[13] Masini E, Carta F, Scozzafava A, et al. Antiglaucoma carbonic anhydrase inhibitors: a patent review. Exp Opin Ther Patents 2013;23:705–16. 10.1517/13543776.2013.794788 23627893

[14] Aggarwal M, Kondeti B, McKenna R. Anticonvulsant/antiepileptic carbonic anhydrase inhibitors: a patent review. Exp Opin Ther Patents 2013;23:717–24. 10.1517/13543776.2013.782394 23514045

[15] Lou Y, McDonald PC, Oloumi A, et al. Targeting tumor hypoxia: suppression of breast tumor growth and metastasis by novel carbonic anhydrase IX inhibitors. Cancer Res 2011;71:3364–76. 21415165 10.1158/0008-5472.CAN-10-4261

[16] Neri D, Supuran CT. Interfering with pH regulation in tumours as a therapeutic strategy. Nat Rev Drug Discov 2011;10:767–77. 21921921 10.1038/nrd3554

[17] Davis RA, Vullo D, Maresca A, et al. Natural product coumarins that inhibit human carbonic anhydrases. Bioorg Med Chem 2013;21:1539–43. 22892213 10.1016/j.bmc.2012.07.021

[18] De Luca L, Mancuso F, Ferro S, et al. Inhibitory effects and structural insights for a novel series of coumarin-based compounds that selectively target human CA IX and CA XII carbonic anhydrases. Eur J Med Chem 2018;143:276–82. 29197732 10.1016/j.ejmech.2017.11.061

[19] Ruiu S, Anzani A, Orrù A, et al. Methoxyflavones from *Stachys glutinosa* with binding affinity to opioid receptors: in silico, *in vitro*, and in vivo studies. J Nat Prod 2015;78:69–76. 25562563 10.1021/np500671v

[20] Fois B, Bianco G, Sonar VP, et al. Phenylpropenoids from *Bupleurum fruticosum* as anti-human rhinovirus species A selective capsid binders. J Nat Prod 2017;80:2799–806. 29039946 10.1021/acs.jnatprod.7b00648

[21] Cerri R, Pintore G, Dessi G, et al. Isolation, characterization and pharmacological activity of *Magydaris pastinacea* (Lam) Paol. glucosides. Farmaco 1995; 50:841–8. 8634074

[22] Rosselli S, Maggio A, Bellone G, et al. Antibacterial and anticoagulant activities of coumarins isolated from the flowers of *Magydaris tomentosa*. Planta Med 2007;73:116–20. 17128388 10.1055/s-2006-951772

[23] Autore G, Marzocco S, Formisano C, et al. Cytotoxic activity and composition of petroleum ether extract from *Magydaris tomentosa* (Desf.) W. D. J. Koch (Apiaceae). Molecules 2015;20:1571–8. 25603502 10.3390/molecules20011571 PMC6272710

[24] Elgamal MHA, Shalaby NMN, Duddeck H, et al. Coumarins and coumarin glucosides from the fruits of *Ammi majus*. Phytochemistry 1993; 34:819–23.

[25] Mohamadi F, Richards NG, Guida WC, et al. MacroModel-an integrated software system for modeling organic and bioorganic molecules using molecular mechanics. J Comput Chem 1990;11:440–67.

[26] Halgren TA. Merck molecular force field. II. MMFF94 van der Waals and electrostatic parameters for intermolecular interactions. J Comput Chem 1996;17:520–52.

[27] Kollman PA, Massova I, Reyes C, et al. Calculating structures and free energies of complex molecules: combining molecular mechanics and continuum models. Acc Chem Res 2000;33:889–97. 11123888 10.1021/ar000033j

[28] Berman HM, Westbrook J, Feng Z, et al. The protein data bank. Nucl Acids Res 2000;28:235–42. 10592235 10.1093/nar/28.1.235 PMC102472

[29] Leitans J, Kazaks A, Balode A, et al. Efficient expression and crystallization system of cancer-associated carbonic anhydrase isoform IX. J Med Chem 2015;58:9004–9. 26522624 10.1021/acs.jmedchem.5b01343

[30] Jorgensen WL. OPLS force fields. In: von Rague Schleyer P, ed. Encyclopedia of computational chemistry. Vol 3. Wiley: Chichester; 1998:1986–1989.

[31] Khalifah RG. The carbon dioxide hydration activity of carbonic anhydrase. J Biol Chem 1971;246:2561–73. 4994926

[32] (a) Bua S, Bozdag M, Del Prete S, et al. Mono- and di-thiocarbamate inhibition studies of the δ-carbonic anhydrase TweCAδ from the marine diatom *Thalassiosira weissflogii*. J Enzyme Inhib Med Chem 2018;33:707–13. 29577755 10.1080/14756366.2018.1450400 PMC6010021

[33] (a) Nocentini A, Bonardi A, Gratteri P, et al. Steroids interfere with human carbonic anhydrase activity by using alternative binding mechanisms. J Enzyme Inhib Med Chem 2018;33:1453–9. 30221552 10.1080/14756366.2018.1512597 PMC7011995

[34] (a) Awadallah FM, Bua S, Mahmoud WR, et al. Inhibition studies on a panel of human carbonic anhydrases with N1-substituted secondary sulfonamides incorporating thiazolinone or imidazolone-indole tails. J Enzyme Inhib Med Chem 2018;33:629–38. 29536779 10.1080/14756366.2018.1446432 PMC6009853

[35] Rosa A, Atzeri A, Nieddu M, et al. New insights into the antioxidant activity and cytotoxicity of arzanol and effect of methylation on its biological properties. Chem Phys Lipids 2017;205:55–64. 28476462 10.1016/j.chemphyslip.2017.05.001

[36] Snatzke G. Circulardichroismus-X: modifizierung der octantenregel für α,β-ungesättigte ketone: cisoide enone, dienone und arylketone. Tetrahedron 1965;21:439–48.

[37] Lin S, Zhang Y, Liu M, et al. Abietane and C20-norabietane diterpenes from the stem bark of *Fraxinus sieboldiana* and their biological activities. J Nat Prod 2010;73:1914–21. 20961093 10.1021/np100583u

[38] Atta-Ur-Rahman Sultana N, Khan MR, et al. Triterpene and coumarins from *Skimmia laureola*. Nat Prod Lett 2002;16:305–13. 12434985 10.1080/10575630290020613

[39] Boyd DR, Sharma ND, Loke PL, et al. Absolute configuration assignment and enantiopurity determination of chiral alkaloids and coumarins derived from O- and C-prenyl epoxides. Chem Commun 2002;21:3070–1. 10.1039/b208978e 12536821

[40] Trani MC, Carbonetti A, Delle Monache G, et al. Dihydrochalcones and coumarins of *Esenbeckia grandiflora* subsp. *brevipetiolata*. Fitoterapia 2004;75:99–102. 14693231 10.1016/j.fitote.2003.08.004

[41] Kikuchi T, Yokoi T, Umemoto K, et al. Constituents of *Scaevola frutescens* (Miller) Krause. Yakugaku Zasshi 1974;94:1616–9. 4476766 10.1248/yakushi1947.94.12_1616

[42] Yoo SW, Kim JS, Kang SS, et al. Constituents of the fruits and leaves of *Euodia daniellii*. Arch Pharm Res 2002;25:824–30. 12510833 10.1007/BF02976999

[43] Bergendorff O, Dekermendjian K, Nielsen M, et al. Furanocoumarins with affinity to brain benzodiazepine receptors *in vitro*. Phytochemistry 1997;44:1121–4. 9055449 10.1016/s0031-9422(96)00703-0

[44] Abou-Elzahab MM, Adam W, Saha-Möller CR. Synthesis of furocoumarin-type potential intercalative alkylating and oxidizing agents of DNA through dimethyldioxirane epoxidation of imperatorin and its derivatives. Liebigs Annalen der Chemie 1992;1992:731–3.

[45] Thongthoom T, Songsiang U, Phaosiri C, Yenjai C. Biological activity of chemical constituents from *Clausena harmandiana*. Arch Pharm Res 2010;33:675–80. 20512464 10.1007/s12272-010-0505-x

[46] Yan R, Shen J, Liu X, et al. Preparative isolation and purification of hainanmurpanin, meranzin, and phebalosin from leaves of *Murraya exotica* L. using supercritical fluid extraction combined with consecutive high-speed countercurrent chromatography. J Sep Sci 2018;41:2092–101. 29385309 10.1002/jssc.201701423

[47] Lv X, Xin X-L, Deng S, et al. Biotransformation of osthole by *Mucor spinosus*. Process Biochem 2012;47:2542–6.

[48] Supuran CT. How many carbonic anhydrase inhibition mechanisms exist? J Enzyme Inhibit Med Chem 2016;31:345–60. 10.3109/14756366.2015.1122001 26619898

[49] Melis C, Distinto S, Bianco G, et al. Targeting tumor associated carbonic anhydrases IX and XII: highly isozyme selective coumarin and psoralen inhibitors. ACS Med Chem Lett 2018;9:725–9. 30034608 10.1021/acsmedchemlett.8b00170 PMC6047168

[50] Rashidi M, Ahmadzadeh A, Ziai SA, et al. Evaluating cytotoxic effect of nanoliposomes encapsulated with umbelliprenin on 4T1 cell line. *In Vitro* Cell Dev Biol-Anim 2017;53:7–11. 27620062 10.1007/s11626-016-0080-7

[51] Hasan M, Genovese S, Fiorito S, et al. Oxyprenylated phenylpropanoids bind to MT1 melatonin receptors and inhibit breast cancer cell proliferation and migration. J Nat Prod 2017;80:3324–9. 29144746 10.1021/acs.jnatprod.7b00853

[52] Rashidi M, Khalilnezhad A, Amani D, et al. Umbelliprenin shows antitumor, antiangiogenesis, antimetastatic, anti-inflammatory, and immunostimulatory activities in 4T1 tumor-bearing Balb/c mice. J Cell Physiol 2018;233:8908–18. 29797576 10.1002/jcp.26814

[53] Zhang L, Sun X, Si J, et al. Umbelliprenin isolated from *Ferula sinkiangensis* inhibits tumor growth and migration through the disturbance of Wnt signaling pathway in gastric cancer. PloSOne 2019;14:e0207169. 10.1371/journal.pone.0207169 PMC6602182 31260453

[54] Farooq S, Shakeel U, Rehman Dangroo NA, et al. Isolation, cytotoxicity evaluation and HPLC-quantification of the chemical constituents from *Prangos pabularia*. PlosOne 2014; 9:e108713. 10.1371/journal.pone.0108713 PMC4196845 25314269

[55] Hitotsuyanagi Y, Kojima H, lkuta Yukio H, et al. Identification and structure-activity relationship studies of osthol, a cytotoxic principle from *Cnidium monnieri*. Bioorgan Med Chem Lett 1996;6:1791–4.

